# Mapping global carbon footprint in China

**DOI:** 10.1038/s41467-020-15883-9

**Published:** 2020-05-07

**Authors:** Yuantao Yang, Shen Qu, Bofeng Cai, Sai Liang, Zhaohua Wang, Jinnan Wang, Ming Xu

**Affiliations:** 1 School of Management and Economics, Beijing Institute of Technology, 100081 Beijing, People’s Republic of China; 2 Center for Sustainable Development and Intelligent Management Research, Beijing Institute of Technology, 100081 Beijing, People’s Republic of China; 3 Center for Energy and Environmental Policy Research, Beijing Institute of Technology, 100081 Beijing, People’s Republic of China; 4 School for Environment and Sustainability, University of Michigan, Ann Arbor, MI 48109-1041 USA; 5 Center for Climate Change and Environmental Policy, Chinese Academy for Environmental Planning, 100012 Beijing, People’s Republic of China; 6 State Key Joint Laboratory of Environment Simulation and Pollution Control, School of Environment, Beijing Normal University, 100875 Beijing, People’s Republic of China; 7 Department of Civil and Environmental Engineering, University of Michigan, Ann Arbor, MI 48109-2125 USA

**Keywords:** Environmental impact, Environmental social sciences

## Abstract

Developing localized climate mitigation strategies needs an understanding of how global consumption drives local carbon dioxide (CO_2_) emissions with a fine spatial resolution. There is no study that provides a spatially explicit mapping of global carbon footprint in China―the world’s largest CO_2_ emitter―simultaneously considering both international and interprovincial trade. Here we map CO_2_ emissions in China driven by global consumption in 2012 at a high spatial resolution (10 km × 10 km) using a detailed, firm-level emission inventory. Our results show that the carbon footprints of foreign regions in China are concentrated in key manufacturing hubs, including the Yangtze River Delta, Pearl River Delta, and North China Plain. Approximately 1% of the land area holds 75% of the global carbon footprint in China. The carbon footprint hotspots in China identified are the key places in which collaborative mitigation efforts between China and downstream parties that drive those emissions.

## Abstract (web summary)

There lacks a spatially explicit mapping of global carbon footprint in China that considers both international and interprovincial trade. Here the authors map the carbon footprints of global regions in China and show the hotspots concentrated in key manufacturing hubs, including the Yangtze River Delta, Pearl River Delta, and North China Plain.

## Introduction

International trade separates greenhouse gas (GHG) emissions from consumption drivers^1–4^. Globally, large shares of products and services are not consumed locally, which leads to considerable embodied emissions driven by global supply chains. This separation between GHG emissions and final consumption, in turn, undermines local mitigation efforts^5^.

Carbon footprint (CF) accounting (i.e., consumption-based accounting) tracks the GHG emissions driven by supply chains and allocates the mitigation responsibilities to final consumers^6–10^. Previous work has linked GHG emissions to final consumption, but primarily at the national^11–15^ or regional^1,16–19^ levels. Given the increasing importance of non-state actors―provinces/states, cities, and companies―in climate mitigation, it becomes increasingly important to spatially explicitly link final consumers to subnational actors that have direct control over GHG emissions.

China, the world’s largest GHG-emitting nation^20^, has long been the primary producer of various industrial and consumer products. A significant share of China’s GHG emissions can be attributed to the final consumption of other nations and regions, given that approximately one-quarter of China’s gross domestic product (GDP) is from exports. Existing studies have investigated global CFs in China at the national level^14^ or at the provincial level considering the interregional trade within China^1^, but without spatially explicit emission profiles. Although global CFs hotspots have been spatially explicitly mapped for nations worldwide including China^21^, interregional trade within China was not considered. Given the heterogeneities of regional economies within China, the CFs of producing the same product can be significantly different across regions^22^. To the best of our knowledge, there is no assessment of spatially explicit CF in China that considers both international and interprovincial trade. The lack of such information can lead to misinterpretation of the linkage between emissions and final consumers. The failure to trace emission drivers along both international and interprovincial supply chains could further obstruct climate mitigation efforts for non-state actors in China.

Here, we spatially explicitly link carbon dioxide (CO_2_) emissions within mainland China to final consumers worldwide through both international and interprovincial trade in 2012. Specifically, we first nest an interprovincial multi-regional input-output (MRIO) model for China^23^ into a global MRIO model^24^. We then link the most recent and detailed spatially explicit CO_2_ emission inventories of China^25^ to the nested MRIO model. These high-quality and fine-scale inventories are derived from large-scale, bottom-up surveys of individual firms in all industries that generate CO_2_ emissions. The resulting CF maps show the locations and magnitudes of a region’s CF in China at a spatial resolution of 10 × 10 km. These detailed CF maps offer insights to guide consumption-based policymaking to avoid carbon leakage and identify emission hotspots for targeted mitigation opportunities for non-state actors in China.

## Results

### Carbon footprint hotspots in China in 2012

**Fig. 1:**
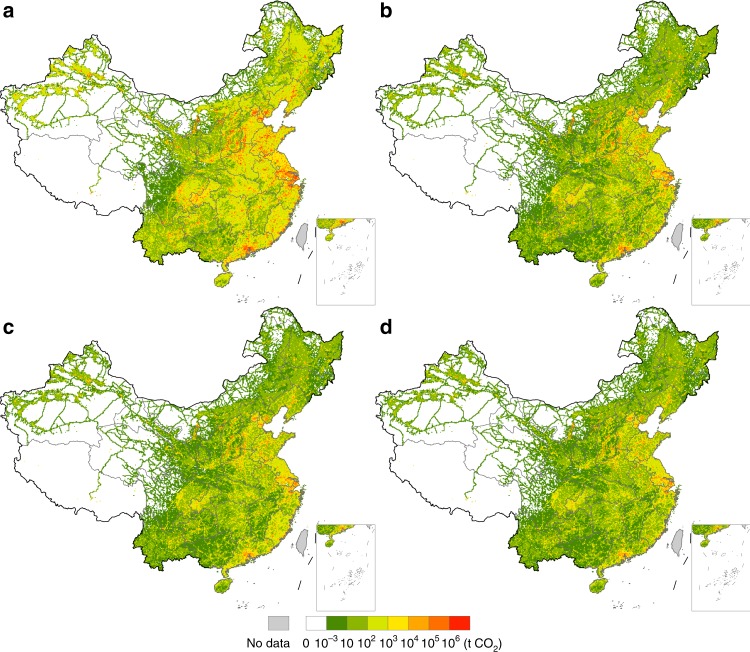
Carbon footprint hotspots of global and regional consumptions in mainland China in 2012. **a** shows carbon footprint (CF) hotspots of foreign final consumption. **b**–**d** show carbon footprint hotspots of the consumption of the United States, Hong Kong, and Japan, respectively. Among all foreign regions, the United States, Hong Kong, and Japan have the largest CFs in China, contributing ~23.0%, 10.8%, and 9.0%, respectively, to the total foreign CF in China in 2012.

The CF hotspot map in Fig. 1a identifies the location of CO_2_ emissions in China driven by foreign final consumption in 2012. The total CF driven by foreign final consumption is 1466 million tonnes (Mt), which accounts for 14.6% of the total industrial-related CO_2_ emissions in China in 2012. This emission volume (1466 Mt) ranks the 5th in the world only after the mainland China, the United States (US), India, and Russia^26^.

The CF hotspots in China driven by global consumption mainly emerge in cities in the Yangtze River Delta (e.g., Shanghai, Ningbo, Suzhou (Jiangsu), Xuzhou, Nanjing), North China Plain (e.g., Tianjin, Tangshan, Beijing, Handan), and Pearl River Delta (e.g., Dongguan, Guangzhou, Foshan). All cities are listed in descending order in terms of their CFs (hereafter). These areas are global hubs of manufacturing and exports for many industrial and consumer products. The emissions from these regions are closely linked with global consumption through downstream supply chains. Additional CF hotspots are scattered across northern, central, and southern China, where key manufacturing industries are located, such as in Ordos, Panzhihua, Linfen, and Pingdingshan.

As China’s largest export destination, the US is also responsible for the largest share of the global CF in China. Hotspots for the US CF in China are located in the Yangtze River Delta notably in the cities of Shanghai, Suzhou, Ningbo, Xuzhou, Pearl River Delta concentrated in the cities of Dongguan, Guangzhou, Foshan, Huizhou, and Jing-Jin-Ji region particularly in the cities of Tianjin and Tangshan (Fig. 1b). All these cities are key manufacturing bases in China, and most of them have or are close to ports for maritime shipping. Exports from these ports drive large amounts of CO_2_ emissions in these cities. Given the pivotal role in trading with China, disaggregated analysis of CF hotspots for the US is presented in a later subsection.

Hong Kong, a special administrative region of China, relies heavily on mainland China for its consumption. Moreover, a large share of China’s exports is re-exported through Hong Kong. As a result, Hong Kong has a large CF in mainland China. Approximately 70% of Hong Kong’s CF in China is driven by household final consumption (44.9%) and gross fixed capital formation (25.7%). The CF hotspots of Hong Kong in mainland China are mainly located in the Yangtze River Delta, particularly in the cities of Shanghai, Ningbo, and Suzhou, and Pearl River Delta, concentrated in the cities of Dongguan, Guangzhou, and Foshan. Additional hotspots can be found in the cities of Tianjin, Tangshan, Beijing, and Ordos (Fig. 1c).

Japan is the world’s third largest economy and China’s second largest export destination just after the US (excluding Hong Kong). Notable CF hotspots of Japan in China are in the cities of Shanghai, Ningbo, and Suzhou in the Yangtze River Delta and are also scattered across the coastal region in the North China Plain (Fig. 1d). Nearly 90% of Japan’s CF in China is driven by household final consumption (63.1%) and gross fixed capital formation (26.2%).

The rest of the ten regions with the largest CFs in China are Germany, Great Britain, South Korea, India, Canada, France, and Italy. The Supplementary Figs. 1–7 provide each region’s CF hotspot map in mainland China.

**Fig. 2:**
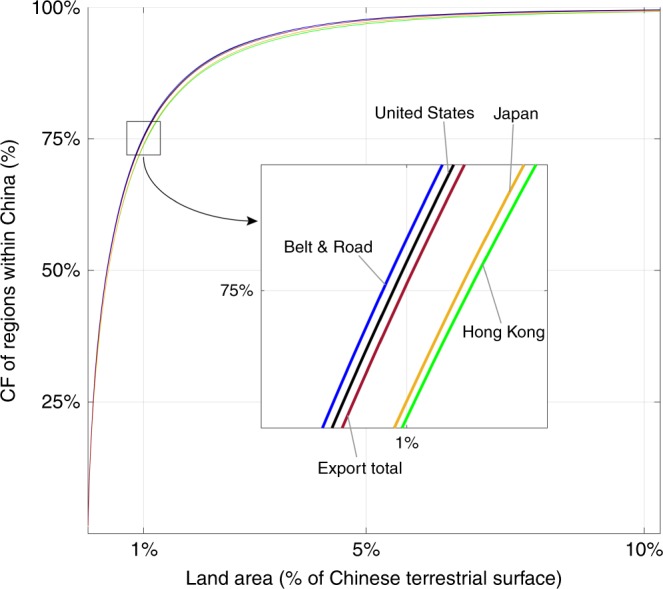
Spatial spread of CF of selected regions in China in 2012. It shows the minimum fraction of the Chinese land area required to hold the CFs of regions. The horizontal axis represents the shares of the land area of the Chinese terrestrial surface, and the vertical axis represents the proportions of CF within China.

Global CF hotspots in China are spatially concentrated in a small area of land. As shown in Fig. 2, only 1% of China’s land area encompasses ~75% of the CF of global consumption. Only slightly >2.2% of the land area in China is needed for 90% of the CF. This relatively small area of land corresponds to the manufacturing hubs in China―Yangtze River Delta, Pearl River Delta, and North China Plain―where CO_2_ emissions occur as a result of producing goods for export.

**Fig. 3:**
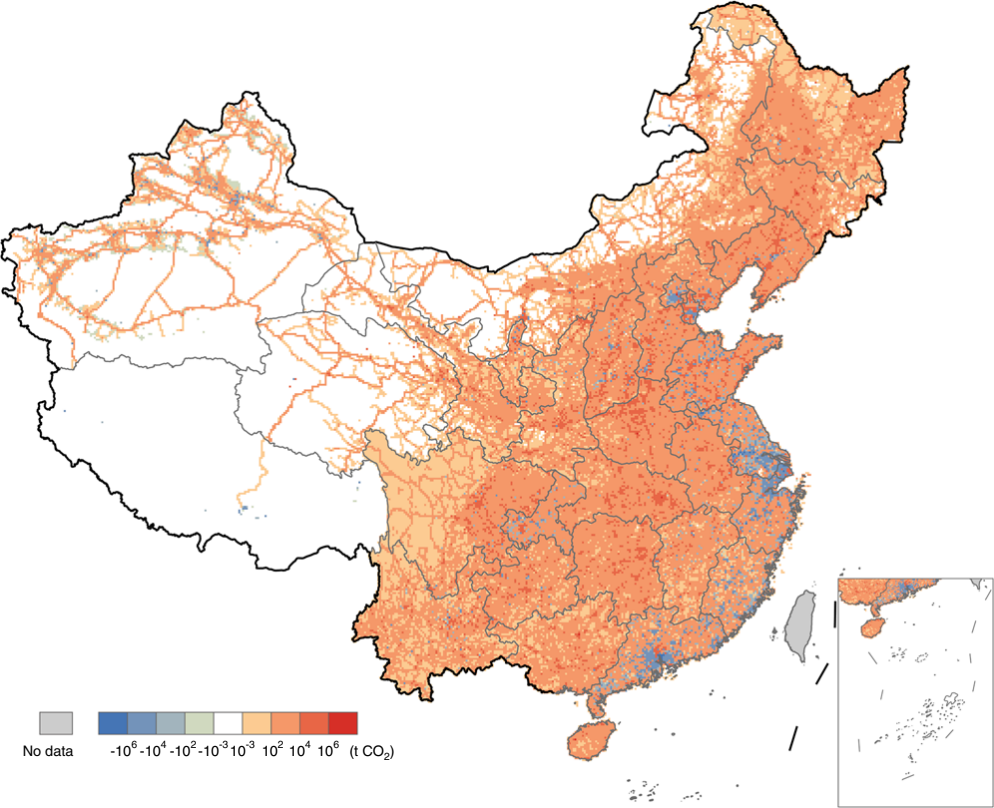
Emission differences between 14.6% of the original CHRED emission map and the export-driven carbon footprint map. Blue grids mean that 14.6% of the original CHRED emissions in these grids are less than the export-driven carbon footprint, while red grids imply the opposite.

The spatial distribution of these export-driven CO_2_ emissions is quite different from that of the simple proportional (i.e., 14.6%) emissions directly obtained from the China High Resolution Emission Database (CHRED). To see this, we map the difference between these two results (the 14.6% of CHRED emission map minus the export-driven CF hotspot result) in Fig. 3. Great differences can be seen in these 10 ×10 km grids ranging from approximately –13.7 Mt in northern Shanghai to ~2.2 Mt in eastern Pingdingshan. The differences mainly locate in cities in the Yangtze River Delta particularly in the cities of Shanghai (37.9 Mt in total absolute difference (the sum of the absolute values of the difference of all the emission grids within a specific city’s boundary) and –33.7 Mt in total actual difference (the sum of the actual values of the difference of those grids)), Ningbo (16.0 and –15.5 Mt), Suzhou (12.6 and –12.4 Mt), and Pearl River Delta notably in the cities of Guangzhou (10.1 and –8.6 Mt), Foshan (8.1 and –8.0 Mt), Huizhou (6.8 and –6.3 Mt). Other notable differential emissions can be seen in the cities of Beijing (7.2 and –3.4 Mt), Pingdingshan (6.0 and 6.0 Mt), Wuhan (4.8 and 4.8 Mt), and so on. These differential hotspots indicate the importance of improving the accuracy of CF analysis within China.

### Carbon footprint hotspots in China by sectors

Among all the exporting sectors, energy-intensive industries are the main contributors. They are production and supply of electricity, steam, gas, and water sector (S22, 42.3%), smelting and processing of metals sector (S14, 13.6%), nonmetallic mineral products sector (S13, 11.5%), petroleum refining, coking, and processing of nuclear fuel sector (S11, 8.7%), and chemical products sector (S12, 7.9%). The contributions of other industries are relatively small, ranging from 0.03% for other manufacture sector (S20) to 3.9% for mining and washing of coal sector (S2).

**Fig. 4:**
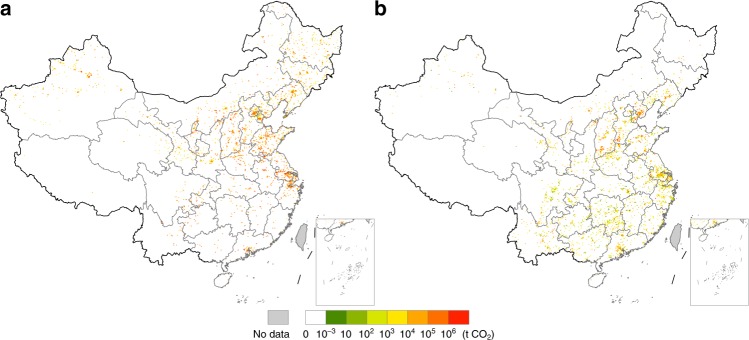
Export-driven carbon footprint hotspots in two different sectors in China. **a** shows the carbon footprint hotspots driven by export in production and supply of electricity, steam, gas, and water sector, and **b** shows the carbon footprint hotspots driven by export in smelting and processing of metals sector.

S22 is the largest CO_2_-emitting sector in China, accounting for 40.2% of the industrial-related CO_2_ emissions in China in 2012. Owing to the resource endowment in China, coal takes the major proportion in the energy mix of power generation, resulting in significant amounts of emissions from it. Also, its emission intensity (CO_2_ emissions per unit output) is the largest among all 25 sectors in 23 out of the 31 provinces, ranging from 0.73 tCO_2_/kUSD in Beijing to 11.76 tCO_2_/kUSD in Inner Mongolia. From the data in China’s MRIO table, we can see that only eight provinces, including Guangdong, Yunnan, Guangxi, Inner Mongolia, Beijing, Jilin, Xinjiang, and Heilongjiang (in descending order of exporting value) exported electricity in 2012 and the exporting value accounts for only 0.06% of the total export^23^. Exports of electricity to countries that are not adjacent to China such as the US and Japan are due to the use of electricity in China by foreign airplanes or vessels. Export-driven CF in S22 in one province of China can be driven directly by the export of S22 product (electricity) and indirectly by the export of other products, causing a significant amount of CF across mainland China (Fig. 4a). The CF hotspots in S22 can be found mainly in the Bohai Rim and Yangtze River Delta concentrated in the cities of Shanghai, Ningbo, and Zhenjiang. Additional hotspots can be found across northern China, especially in the northwestern and northeastern regions where many power plants are located.

China is the largest steel producer in the world as well as the largest exporter^27^. Exports lead to notable CF hotspots in S14 in northern Shanghai, and the cities of Tangshan and Handan in Hebei Province, Linfen and Yuncheng in Shanxi Province, and Anshan and Benxi in Liaoning Province (Fig. 4b). These cities are the main manufacturing bases of iron and steel that use a large amount of coal. Unlike the CF hotspots in S22 that driven by exports, many CF hotspots in S14 caused by exports are also scattered across southern China.

CF hotspots in S13, S11, and S12 in China that driven by exporting products show different spatial distribution patterns (see Supplementary Fig. 8a–c). The CF hotspots in S13 in China caused by exports are mainly distributed below the Heihe-Tengchong Line, notably in Qingyuan, Foshan, Zhaoqing, and Huizhou in Guangdong Province and Longyan in Fujian Province (Supplementary Fig. 8a). Among them, Qingyuan, Huizhou, and Longyan are three of the top ten origins for cement production. The CF hotspots in S11 driven by exports are concentrated in Shanghai, Tangshan, Tianjin, and the central region of the North China Plain (Supplementary Fig. 8b), where some large refineries are located. Additional hotspots can be found in Dalian, Guangzhou, and Ningbo. For S12, the export-driven CF hotspots are located in the Yangtze River Delta and Pearl River Delta in the cities of Shanghai, Huizhou, Yangzhou, and Zhenjiang (Supplementary Fig. 8c).

### Carbon footprint hotspots in China by final demand categories

Among the six categories of final demand (see Methods), household final consumption drives approximately half of the total CF of Chinese exports (48.4%), followed by gross fixed capital formation (28.5%). The majority of Chinese exports are consumer products and intermediate products used to produce consumer products in other regions. Therefore, household final consumption is the largest driver of the global CF in China. In contrast, China’s domestic final demand is dominated by gross fixed capital formation due to rapid infrastructure development and urbanization. As a result, the CF of Chinese domestic consumption is largely affected by gross fixed capital formation (65.1%), followed by urban household consumption (19.5%) and rural household consumption (6.2%).

**Fig. 5:**
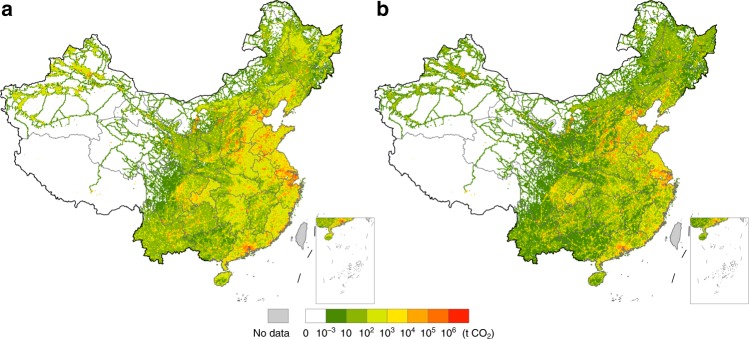
Carbon footprint hotspot maps driven by exports for two different categories of final demand. **a** shows the carbon footprint hotspots driven by exports for household consumption, and **b** shows the carbon footprint hotspots driven by exports for gross fixed capital formation.

The CF hotspots of global household final consumption are mostly located in cities of the Yangtze River Delta and Pearl River Delta, such as Shanghai, Ningbo, Suzhou, Dongguan, and Guangzhou (Fig. 5a). The CF hotspots of global gross fixed capital formation are primarily in the Yangtze River Delta, including the cities of Shanghai, Suzhou, Ningbo, and Nanjing. Additional hotspots can be observed in the cities of Anshan, Pingdingshan, Shaoguan, Panzhihua, and Benxi, which are key hubs of steel production in China (Fig. 5b).

### Carbon footprint hotspots of the United States

In 2012, the US was the largest driver of CO_2_ emissions in China (337 Mt), nearly half of which were driven by its household final consumption (49.2%), followed by gross fixed capital formation (17.1%), non-profit institutions serving households (11.9%), government final consumption (9.1%), changes in inventories (7.5%), and acquisitions less disposals of valuables (5.2%). As a highly industrialized and the largest economy in the world, the US’ infrastructure and machinery development are more sophisticated^28^_,_ and the proportion of gross fixed capital formation in GDP is lower than that of other nations^29^, resulting in relatively low proportion emissions that caused by gross fixed capital formation. At the aggregated level, the proportion of CF driven by capital formation (including gross fixed capital formation and changes in inventories) is close to those from Hong Kong (25.7% + 1.8%) and Japan (26.2% + 0.1%).

In 2012, the US imported 22.3 billion USD worth of products mainly from high-tech and heavy industries, including electronic equipment and measuring instruments sector (S19, 60.6%) and general- and special-purpose machinery sector (S16, 15.9%) from Guangdong, Jiangsu, Shanghai, and Zhejiang for gross fixed capital formation. Meanwhile, the US imported 5.5-fold that of products mainly from S19 (29.6%), textile wearing apparel, leather, fur, and its products sector (S8, 22.4%), and papermaking, printing, stationery, etc. sector (S10, 17.2%) from Guangdong, Jiangsu, Shanghai, Zhejiang, and Fujian for household final consumption^23,24^, most of which is less carbon-intensive. However, the CF in China caused by household final consumption is only 2.9-fold caused by gross fixed capital formation, which implies that the life cycles of products for capital formation, such as infrastructure development and machinery, tend to be more carbon-intensive than those of consumer goods for the household. Upgrading domestic production technologies requires capital formation, which may lead to significant carbon emissions along the global supply chains^30^. It is crucial to manage such carbon leakage with improved information.

**Fig. 6:**
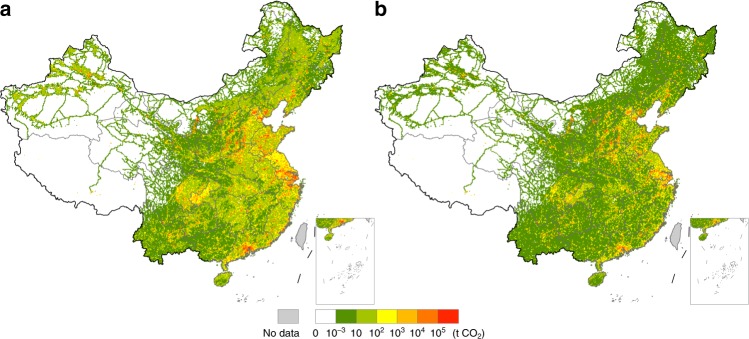
Carbon footprint hotspot maps driven by two different categories of final demand of the United States. **a** shows the carbon footprint hotspots driven by household consumption, and **b** shows the carbon footprint hotspots driven by gross fixed capital formation.

The CF hotspots caused by household final consumption of the US mostly arise in the cities of Shanghai, Ningbo, Dongguan, Suzhou, Jiaxing, Pingdingshan, Zhenjiang, and Taizhou (Zhejiang) (Fig. 6a). Take Shanghai that has a large manufacturing sector despite >50% of its GDP from the service sector. Approximately 50% of its industrial output is from carbon-intensive heavy industries, making Shanghai the top CO_2_-emitting city in China^31^, and accounting for 2.09% of national total. Other hotspot cities are dominated by light manufacturing or high-tech industries that produce large amounts of consumer products for both domestic consumption and export.

At the sector level, the largest CF driven by the US household final consumption predominately comes from S22 (42.7%). The associated CF hotspots are mostly located in the cities of Ningbo, Shanghai, Suzhou, and Xuzhou (Supplementary Fig. 9a). S14 contributes to 11.8% of the US household CF in China, with key hotspots in the cities of Shanghai, Tangshan, Handan, Nanjing, Suzhou, Anshan, Shaoguan, and Benxi (Supplementary Fig. 9b). Notably, the CF hotspots of S13 (11.2%) are not among those hotspots that have frequently been identified above, including the cities of Qingyuan, Chongqing, Longyan, Foshan, and Zhaoqing (Supplementary Fig. 9c).

The spatial distribution of CF hotspots driven by the US gross fixed capital formation shows differences: these hotspots are mainly located in Shanghai, Suzhou, Tangshan, and Tianjin (Fig. 6b). Shanghai is one of the top-emitting cities in China (2.09% of national total) and has already been identified in CF hotspots analysis for household final consumption. Suzhou and Tianjin are leading high-tech industrial cities in China and produce significant amounts of emissions (1.13% and 1.56% of national total, respectively). Tangshan is a world-famous heavy industry city for its commanding lead in iron and steel production, making it another top CO_2_-emitting city in China (1.81% of national total). These cities together provide materials and products for electronic equipment and machinery production in coastal China which are in turn exported for global capital formation. Apart from those hotspot cities also identified for the household final consumption CF, hubs of iron and steel manufacturing, such as Anshan, Pingdingshan, and Panzhihua, are also hotspot cities for the gross fixed capital formation CF.

From the sectoral perspective, the CF from S22 accounts for 41.3% of the total CF driven by the US gross fixed capital formation. The exports for the US gross fixed capital formation are mainly products from S19 and S16 and the production of these high-tech and heavy equipment and machinery requires great amounts of electricity. The hotspots mainly emerge in the North China Plain and the Yangtze River Delta, including the cities of Shanghai, Suzhou, Xuzhou, and Ningbo (Supplementary Fig. 9d). The sectors S14 and S13 contribute 17.0% and 16.4%, respectively. The CF hotspot distributions for these two sectors are quite similar to the aforementioned distribution of household final consumption CF (Supplementary Fig. 9e, f).

## Discussion

Through this study, we show that most of the CF of China’s export occurs in a small number of hubs occupying a small portion of land area in China. Our results for 2012 show that 14.6% of China’s industrial-related CO_2_ emissions were driven by foreign final consumption. The CF hotspots are mainly located in the Yangtze River Delta, Pearl River Delta, and North China Plain, notably in the cities of Shanghai, Ningbo, Suzhou (Jiangsu), Tianjin, Tangshan, Xuzhou, Nanjing, and Dongguan. The US, Hong Kong, and Japan are the top three regions with the largest CFs in China. Overall, 1% of land area in China can serve ~75% of global CFs, while 90% of global CFs need only slightly >2.2% of the land area. Approximately 42% of the export-driven CO_2_ emissions in China are emitted by the electricity industry with notable CF hotspots in the cities of Shanghai, Ningbo, Suzhou, and Xuzhou. Meanwhile, over 10% of the total global CF in China has arisen from both steel and cement industries. The export-driven CF hotspots in the steel industry mainly emerge in the cities of Shanghai, Tangshan, Handan, Nanjing, and Anshan, while exports generate CF hotspots in the cement industry mainly in the Pearl River Delta.

From the perspective of economic theory, the optimal abatement strategy for CO_2_ emissions would simply equalize the marginal abatement costs. In the real world, transaction costs, including acquiring reliable information and drafting detailed contracts, prevent this ideal situation^10^. Emission mitigation actions could exert adverse social impacts, such as habitability changes and poverty. In some areas, these actions could decrease employment due to the shutdown of manufacturing enterprises. On the other hand, the internationally transferred mitigation outcomes, as specified in Article 6(2) of the Paris Agreement, recognize nations’ contributions to reduce carbon emissions outside their borders^32^. Consumers and intermediate producers may be willing to clean up the carbon emissions along their supply chains for their consumption, which could even add to nationally determined contributions, but lack the relevant information. The spatially explicit CF maps provide more accurate and detailed local assessments with sector- and category-wise results, which can benefit the collaborative policymaking for localized mitigation strategies.

The spatially explicit CF analysis contributes to sustainable production, interregional and international trade, and consumption^33^. Responsibility for the great amount of CO_2_ emissions (1466 Mt) driven by China’s export should be shared along the international supply chains but not exclusively on emitting industries in China or on global final consumers. The CF maps are more actionable than a proportional emission map of the total emissions, for both producers in China and consumers abroad, to initiate collaborative mitigation efforts on targeted CF hotspots.

Given the current US-China trade dispute, reduced exports from China to the US are expected for goods that are affected by US tariffs. As a result, the CF hotspot map for China’s export to the US will change accordingly. Approximately half (50.2%) of tariffs that are currently imposed on imports from China are related to electromechanical equipment; and the levy on furniture and lighting, base metal, and transportation equipment account for 12.0%, 6.9%, and 6.2%, respectively^34^. As a result, the CF of the US in China will likely decline accordingly in the cities of Chongqing, Ningbo, Shanghai, Langfang, etc.

China’s Belt and Road Initiative is expected to strengthen the trade relationship between China and the Belt and Road nations (~140 nations until Jan 2020^35^), which are mostly located in the Eurasia continent, especially for the ASEAN (Association of Southeast Asia Nations). Our results for 2012 show that the CF hotspots of the Belt and Road nations are widely spread in eastern, central, and northern China (Supplementary Fig. 10). In recent years, the export from China to Belt and Road partner nations mainly focuses on mechanical and electrical equipment and material for transportation infrastructure (i.e., highway, railway, ports, etc.) and energy infrastructure. In the future, it may shift to the export of high-quality production capacity such as high-ending manufacturing, information technology, internet applications, etc. Therefore, these expected increases in exports from China will create more demands for electrical machinery, electronic equipment, etc., and the CF will possibly increase accordingly in the cities of Kunming, Nanjing, Ningbo, etc. Mitigation strategies need to be in place to face the anticipated emission increase in these places.

Uncertainties exist due to inherent assumptions of the MRIO model^36–38^. China is a vast country with multiple industrial clusters and economic zones, some of which are supported by foreign direct investments (FDI) to produce exported commodities. Therefore, nations may have different interregional supply chains within China, leading to different spatial distributions of emission hotspots induced by the same category of exported commodities. In this study, we disaggregate and allocate the export of each product category in each province to the exporting regions assuming that the distribution shares are the same as the corresponding ones at the national level. This assumption may not be consistent with reality and is a limitation of the approach applied here, which results from data unavailability. On the other hand, China’s exports are mainly through the developed eastern coast regions that are equipped with many world-class ports^39^. The primary driving force of international trade is low transportation costs of ocean shipping^40^, and the transportation costs for imports from China’s coastal regions should have little difference. Under this perspective, our assumption on provincial distribution shares of exports seems reasonable. In the future, international trade data on provincial exports by destination and imports by origin can greatly improve the results. Even the integration of China’s interprovincial MRIO model with a global MRIO model represents a significant improvement over the previous investigation on spatially explicit CFs in China, more accurate estimations based on an inter-city input-output model may still be desirable. Currently, such a model does not exist, and its development can provide a critical contribution to the measurement of global CFs in China.

With the same approach, spatially explicit hotspots of other air pollutants and broadly other environmental impacts can also be identified. Future work may also examine the temporal and spatial dynamics of global environmental footprint hotspots in China. Such information has become increasingly needed for non-state actors to participate in global mitigation of various environmental impacts.

## Methods

### Linking the China MRIO model to the Eora MRIO model

The 2012 MRIO model for China is in a 31-province and 42-sector format^23^. The global MRIO model for 2012 is from Eora^24,37^ with 190 nations/regions and 14,839 sectors. The spatially explicit gridded CO_2_ emissions maps for all two-digit International Standard Industrial Classification sectors in 2012, with a resolution of 10 ×10 km, are from the China High Resolution Emission Database (CHRED)^25,41^.

We disaggregate the Chinese portion in Eora into a 31-province table according to the China MRIO model. To keep the spatial emission data of each sector consistent with the economic sectors in the China MRIO model, we first aggregate the China MRIO model and emissions data into a 31-province and 25-sector format (Supplementary Table 1). Based on the original Eora’s export and import proportions for China, the export and import for each sector in each province in the China MRIO model are disaggregated by the remaining 189 nations/regions. We assume that the international exports (or imports) of each sector in a province are distributed among all global regions in the same proportions as those of China’s total exports (or imports). Altogether, the updated global MRIO model contains 189 regions with the same sectors as those in the original Eora model and 31 Chinese provinces with 25 sectors in each province. Previous research on the impacts of trade on China’s carbon emissions has embedded the China MRIO model into the global MRIO model using the same approach^1,17,18^. Theoretically, this method of combining different MRIO models is equivalent to assembling a network and has the properties of transparency, modularity, and efficiency^42^. However, the quantification of global carbon footprint can be further improved once data on country-specific regional heterogeneity of supply chains become available.

The final demand categories of global regions include household final consumption, non-profit institutions serving household, government final consumption, gross fixed capital formation, changes in inventories, and acquisition less disposals of valuables^24^. For Chinese provinces, final demand is categorized into rural household consumption, urban household consumption, government consumption, gross fixed capital formation, and stock change^23^.

### Mapping global carbon footprint in China

We first conduct an environmentally extended MRIO analysis to trace embodied CO_2_ emissions to final consumption along the global supply chains. The basic linear equations of the MRIO model are

1 $$\bf{x} = \left( {I - A} \right)^{ - 1}Y = LY$$

2 $$\begin{array}{l}{\ \ }{\mathbf{x}} = \left[ {\begin{array}{*{20}{c}} {{\mathbf{x}}^1} \\ {{\mathbf{x}}^2} \\ \vdots \\ {{\mathbf{x}}^n} \end{array}} \right],{\mathbf{L}} = \left( {{\mathbf{I}} - \left[ {\begin{array}{*{20}{c}} {{\mathbf{A}}^{11}} & {{\mathbf{A}}^{12}} & \cdots & {{\mathbf{A}}^{1n}} \\ {{\mathbf{A}}^{21}} & {{\mathbf{A}}^{22}} & \cdots & {{\mathbf{A}}^{2n}} \\ \vdots & \vdots & \ddots & \vdots \\ {{\mathbf{A}}^{n1}} & {{\mathbf{A}}^{n2}} & \cdots & {{\mathbf{A}}^{nn}} \end{array}} \right]} \right)^{ - 1}\\ \;\; = \left[ {\begin{array}{*{20}{c}} {{\mathbf{L}}^{11}} & {{\mathbf{L}}^{12}} & \cdots & {{\mathbf{L}}^{1n}} \\ {{\mathbf{L}}^{21}} & {{\mathbf{L}}^{22}} & \cdots & {{\mathbf{L}}^{2n}} \\ \vdots & \vdots & \ddots & \vdots \\ {{\mathbf{L}}^{n1}} & {{\mathbf{L}}^{n2}} & \cdots & {{\mathbf{L}}^{nn}} \end{array}} \right],Y = \left[ {\begin{array}{*{20}{c}} {\mathop {\sum}\nolimits_t {{\mathbf{y}}^{1t}} } \\ {\mathop {\sum}\nolimits_t {{\mathbf{y}}^{2t}} } \\ \vdots \\ {\mathop {\sum}\nolimits_t {{\mathbf{y}}^{nt}} } \end{array}} \right]\qquad\end{array}$$

where ${\mathbf{x}} = \left( {x_j^s} \right)$ is the total output vector in which $x_j^s$ is the total output of sector *j* in region *s*; ${\mathbf{A}}^{rs} = \left( {a_{ij}^{rs}} \right)$ is the technical coefficient submatrix given by $a_{ij}^{rs} = z_{ij}^{rs}/x_j^s$, in which $z_{ij}^{rs}$ is the intersectoral monetary flow from sector *i* in region *r* to sector *j* in region *s*; ${\mathbf{L}} = \left( {{\mathbf{I}} - {\mathbf{A}}} \right)^{ - 1}{\mathrm{ = }}\left( {l_{ij}^{rs}} \right)$ is the Leontief inverse matrix in which $l_{ij}^{rs}$ capture both direct and indirect inputs from sector *i* of region *r* to satisfy one unit of final demand in monetary value in sector *j* in region *s*, and **I** is the identity matrix^43^; ${\mathbf{Y}} = \left( {y_j^{st}} \right)$ is the final demand matrix in which $y_j^{st}$ is the final demand of region *t* for products of sector *j* from region *s*.

The CO_2_ emissions embodied in the products of a sector in a specific region can be calculated by extending the MRIO model with an emission intensity vector ${\mathbf{f}}$, entries in which, $f_i^r$, show the CO_2_ emissions per unit of economic output for sector *i* in region *r*:

3 $${\bf{C}} = {\hat {\bf{f}}} \bf{LY}$$

The figures presented in this paper are the CF hotspots in China driven by the final demand of region *t*:

4 $$H^t = \mathop {\sum}\limits_i {M_i^r} \frac{{f_i^r\mathop {\sum}\limits_{js} {l_{ij}^{rs}y_j^{st}} }}{{d_i^r}}$$

where $M_i^r$ is the gridded emission map term showing the CO_2_ emissions of sector *i* in each grid in region *r*, and $d_i^r$ is the total CO_2_ emissions of sector *i* in region *r*. The embodied emissions term $\bf{fLY}$ that driven by final demand of region *t* in one sector in a specific region is normalized by the total emissions from the same sector of this region ($d_i^r$), so that the CF hotspots $H^t$ are in absolute values that are consistent with *M*. As for the detailed derivation process, Supplementary Data 1 shows the spatial footprint approach.

As we focus on the actual emissions and CFs occurring in China’s territory, we set the emission intensity of all sectors of all foreign regions as 0 in the calculation.

### Reporting summary

Further information on research design is available in the Nature Research Reporting Summary linked to this article.

## Supplementary information

Supplementary Information

Reporting Summary

Description of Additional Supplementary Files

Supplementary Data 1

## Data Availability

The 2012 China MRIO table of 31 provincial units is compiled by the Key Laboratory of Regional Sustainable Development Analysis and Simulation, Chinese Academy of Sciences^23^. The 2012 Full Eora model can be obtained from the Eora Global Supply Chain Database website (https://worldmrio.com/)^24, 37^. The spatially explicit gridded CO_2_ emissions of the China High Resolution Emission Database can be obtained from our previous study^25^. All datasets generated during this study are available from the corresponding author upon reasonable request.

## Code availability

All computer codes generated during this study are available from the corresponding author upon reasonable request.
