# Supplementary material for: Mapping global carbon footprint in China
Source: Nat Commun. 2020 May 7;11:2237. doi: 10.1038/s41467-020-15883-9 (PMC7206023; doi:10.1038/s41467-020-15883-9)
Supplement: Supplementary file 1 — Supplementary Information [file 41467_2020_15883_MOESM1_ESM.pdf]

Supplementary Information

Mapping global carbon footprint in China

Yang et al.

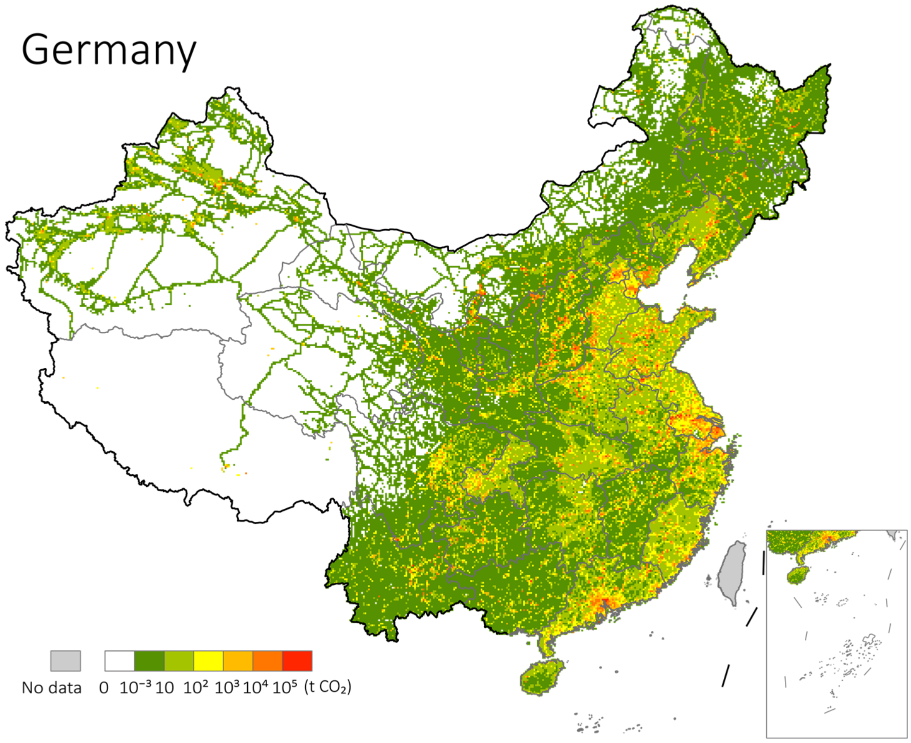

Supplementary Fig. 1 Carbon footprint hotspots of consumption of Germany in mainland China in 2012.

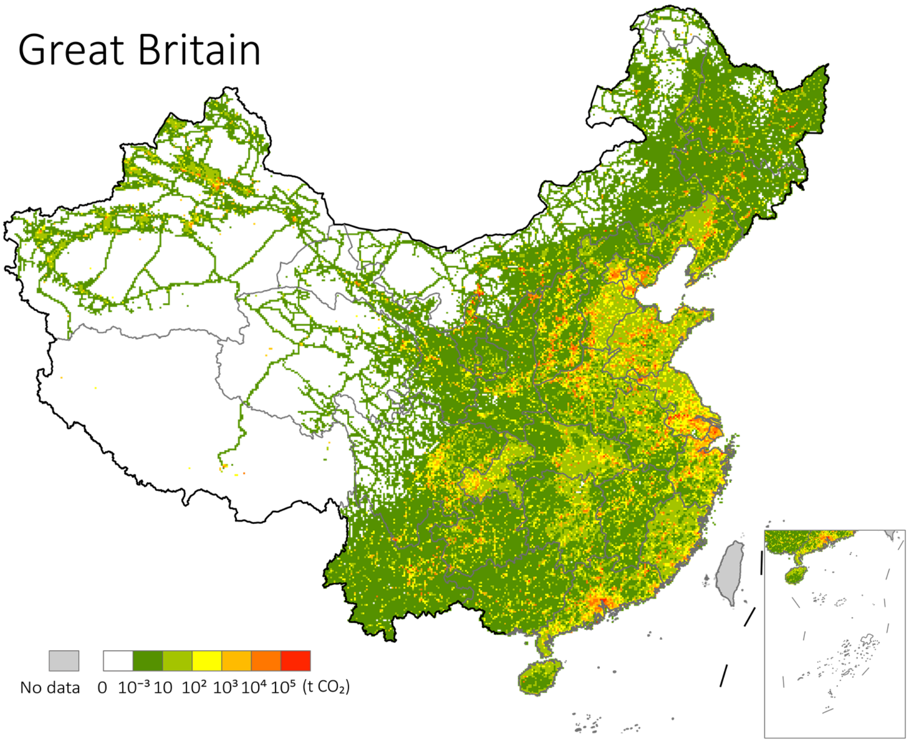

Supplementary Fig. 2 Carbon footprint hotspots of consumption of Great Britain in mainland China in 2012.

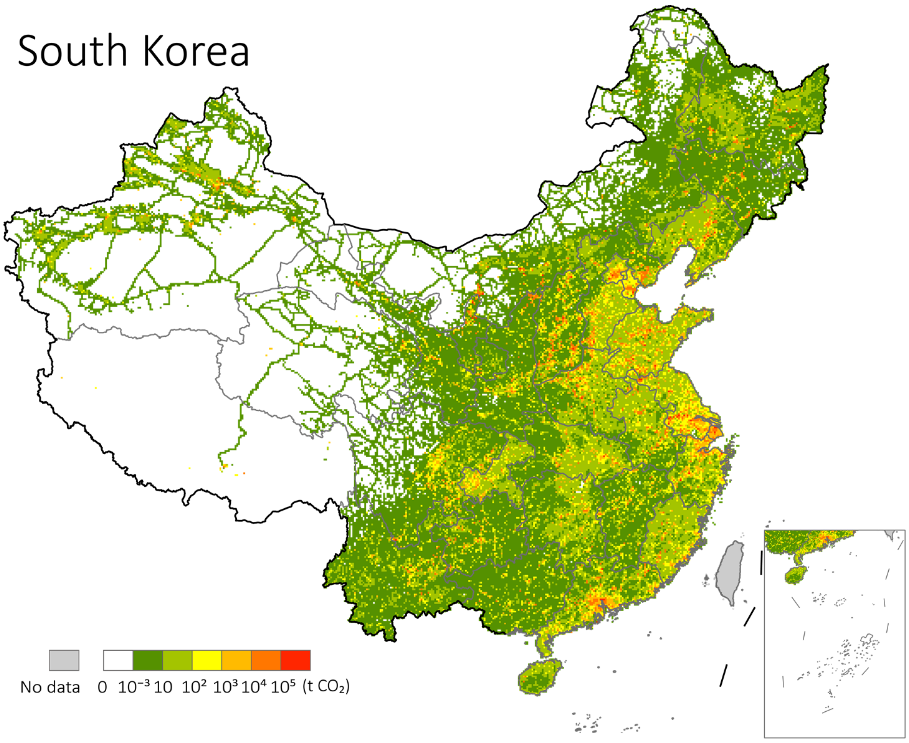

Supplementary Fig. 3 Carbon footprint hotspots of consumption of South Korea in mainland China in 2012.

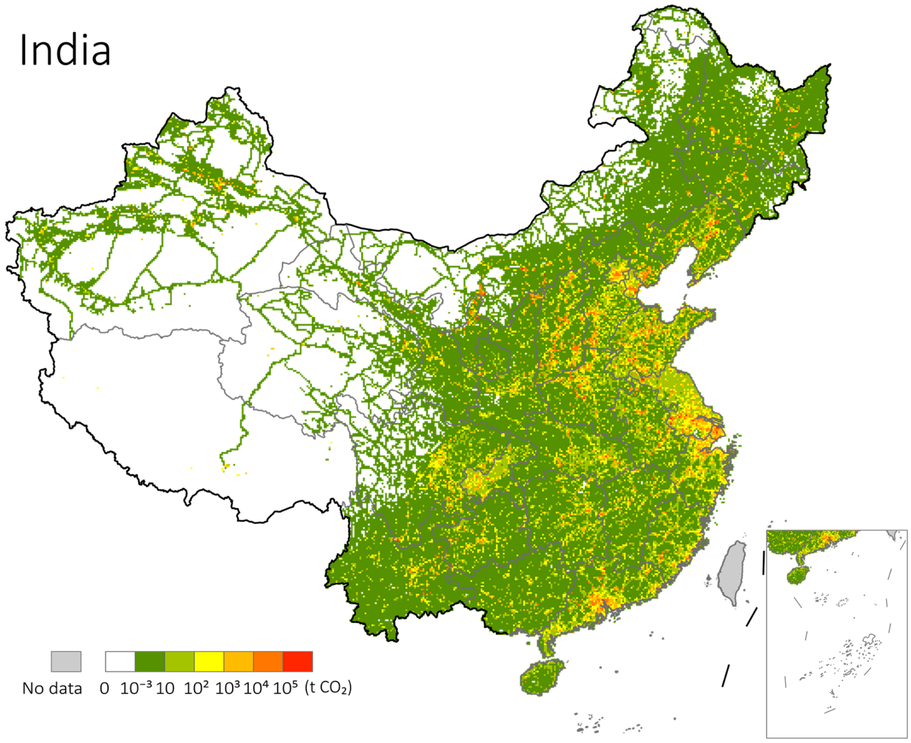

Supplementary Fig. 4 Carbon footprint hotspots of consumption of India in mainland China in 2012.

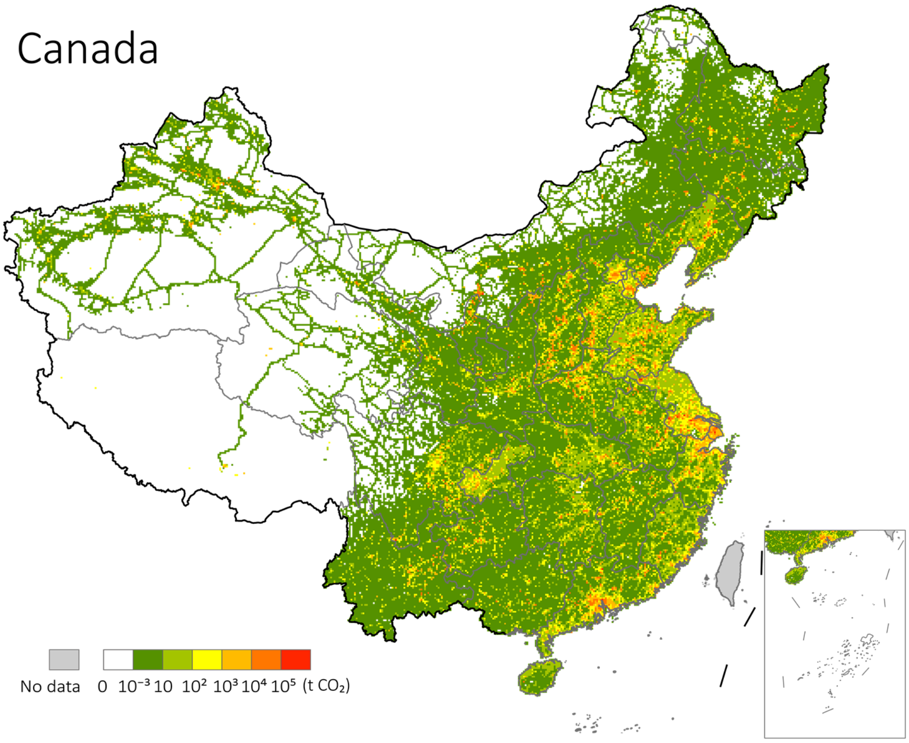

Supplementary Fig. 5 Carbon footprint hotspots of consumption of Canada in mainland China in 2012.

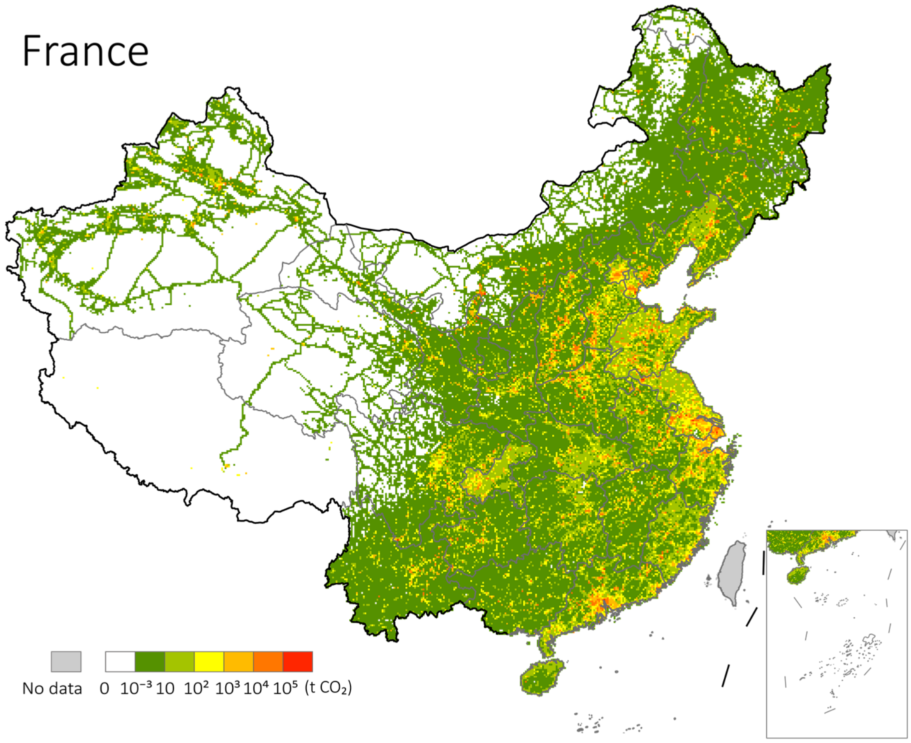

Supplementary Fig. 6 Carbon footprint hotspots of consumption of France in mainland China in 2012.

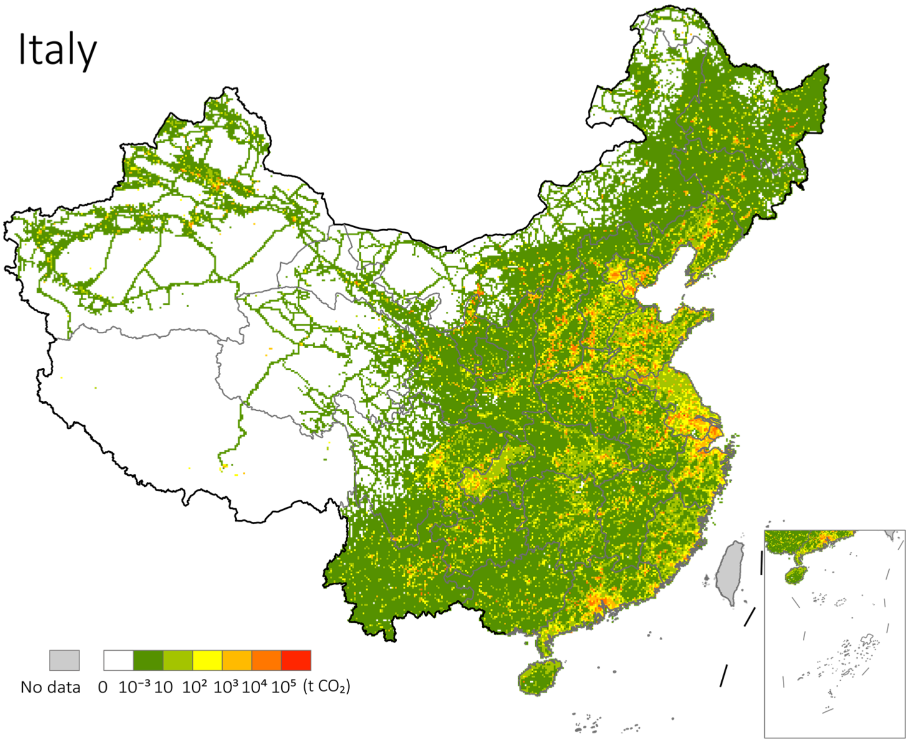

Supplementary Fig. 7 Carbon footprint hotspots of consumption of Italy in mainland China in 2012.

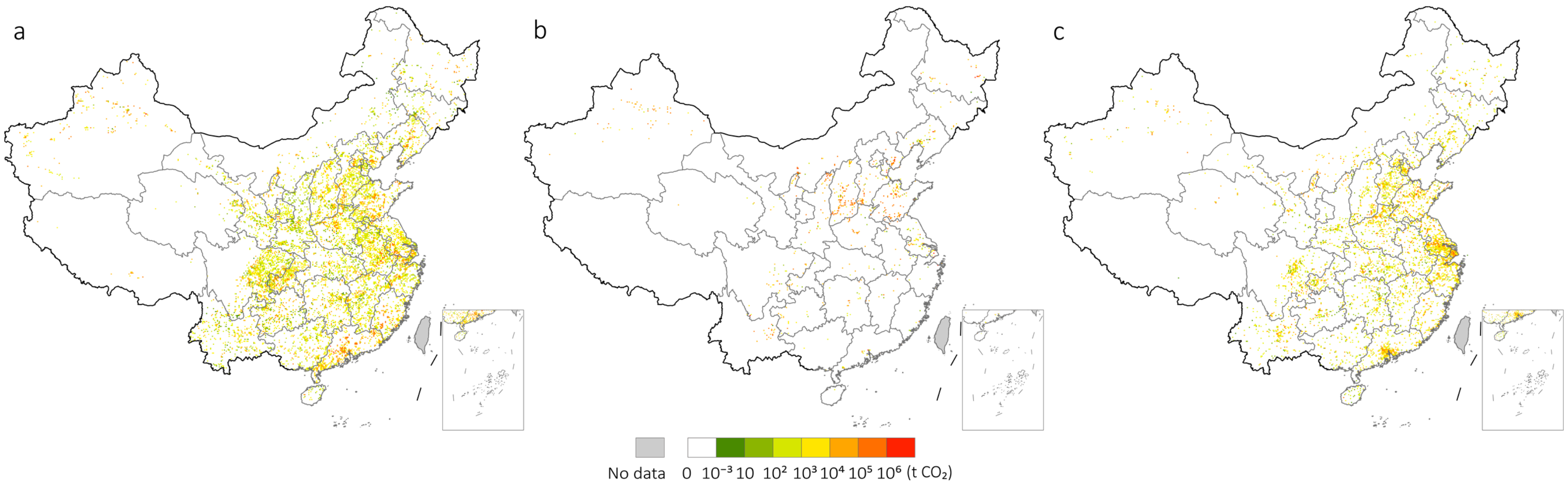

Supplementary Fig. 8 Export-driven carbon footprint hotspots in different sectors in China. Panel **a** shows the hotspots driven by export in nonmetallic mineral products sector, and panel **b** and **c** show the hotspots driven by export in petroleum refining, coking, and processing of nuclear fuel sector and chemical products sector, respectively.

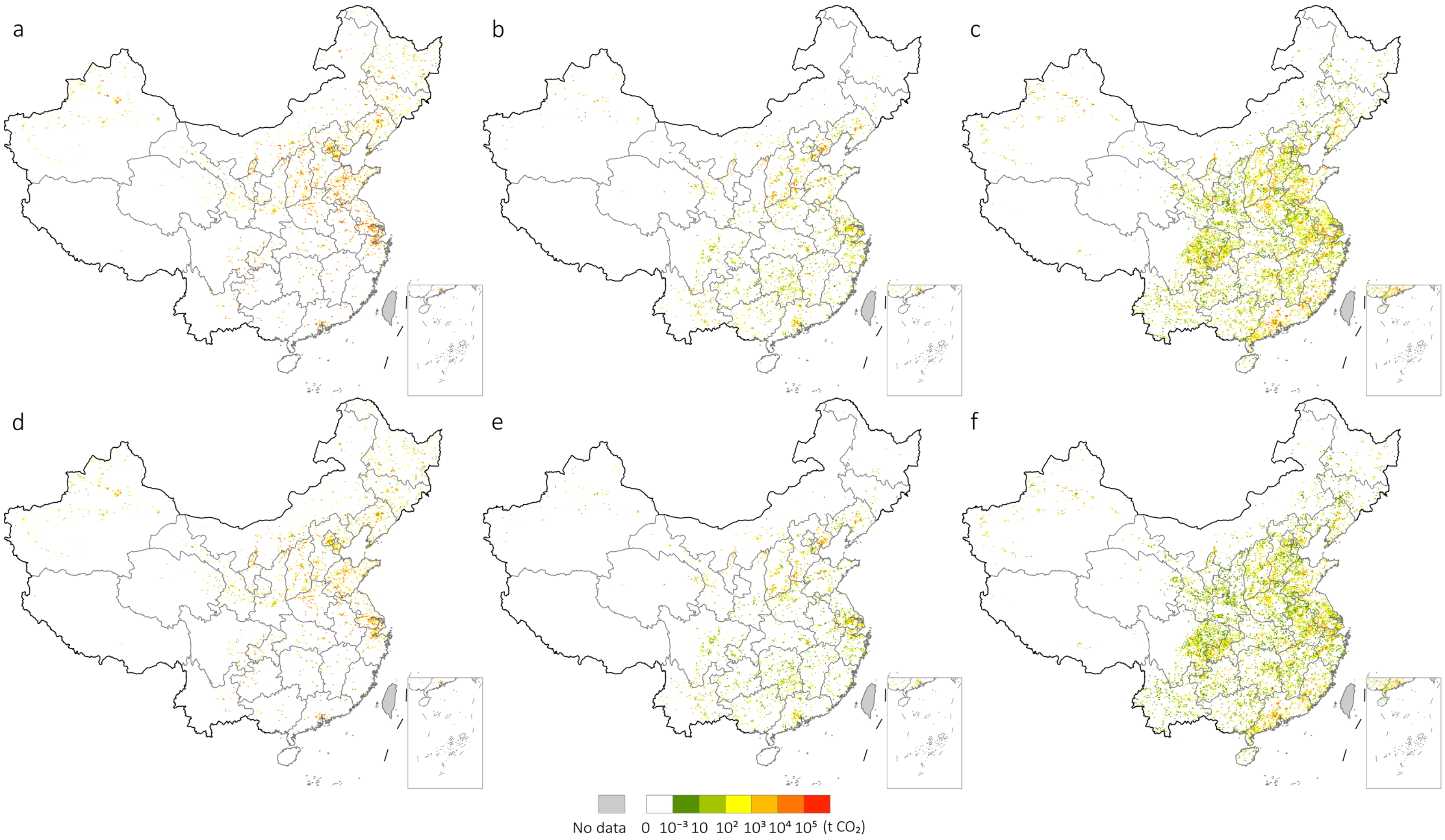

Supplementary Fig. 9 Carbon footprint hotspot maps by sector driven by exports for household final consumption and gross fixed capital formation of the United States. Panels in the upper row indicates carbon footprint hotspots in China that driven by export for household final consumption of the United States in production and supply of electricity, steam, gas, and water sector (a), smelting and processing of metals sector (b), and nonmetallic mineral products sector (c). Panels in the lower row indicates carbon footprint

hotspots in China that driven by export for gross fixed capital formation of the United States in production and supply of electricity, steam, gas, and water sector (d), smelting and processing of metals sector (e), and nonmetallic mineral products sector (f).

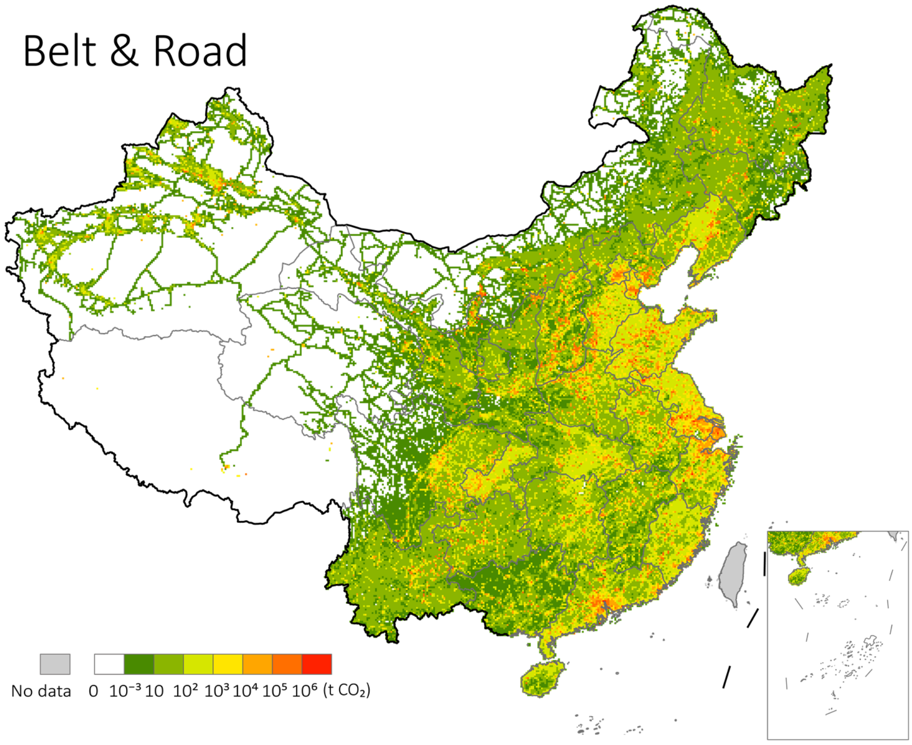

Supplementary Fig. 10 Carbon footprint hotspots of consumption of Belt and Road partner nations in mainland China in 2012.

Supplementary Table 1. Sector classification of each province in the 2012 China multiregional input-output (MRIO) table

Sector code	Original sectors	Sector code	Aggregated sector
1	Farming, forestry, animal production, and fishery	S1	Agriculture
2	Mining and washing of coal	S2	Mining and washing of coal
3	Extraction of crude petroleum and natural gas	S3	Extraction of crude petroleum and natural gas
4	Mining of metal ores	S4	Mining of metal ores
5	Mining of nonmetallic mineral and other mineral	S5	Mining of nonmetallic mineral and other mineral
6	Food and tobacco	S6	Food and tobacco
7	Textiles	S7	Textiles
8	Textile wearing apparel, leather, fur, and its products	S8	Textile wearing apparel, leather, fur, and its products
9	Timbers and furniture	S9	Timbers and furniture
10	Papermaking, printing, stationery, etc.	S10	Papermaking, printing, stationery, etc.
11	Petroleum refining, coking, and processing of nuclear fuel	S11	Petroleum refining, coking, and processing of nuclear fuel
12	Chemical products	S12	Chemical products
13	Nonmetallic mineral products	S13	Nonmetallic mineral products
14	Smelting and processing of metals	S14	Smelting and processing of metals
15	Metal products	S15	Metal products
16	General-purpose machinery	S16	General- and special-purpose machinery
17	Special-purpose machinery		
18	Transport equipment	S17	Transport equipment
19	Electrical machinery and apparatus	S18	Electrical machinery and apparatus
20	Communication equipment, computer, and other electronic equipment	S19	Electronic equipment and measuring instruments
21	Measuring instruments		
22	Other manufacture	S20	Other manufacture
23	Scrap and waste	S21	Scrap and repair of equipment
24	Repair of metal products, machinery, and equipment		
25	Production and supply of electricity and steam	S22	Production and supply of electricity, steam, gas, and water
26	Production and supply of gas		

27	Production and supply of water		
28	Construction	S23	Construction
29	Wholesale and retail trade	S25	Other services
30	Transport, storage, and post	S24	Transport, storage, and post
31	Accommodation and catering	S25	Other services
32	Information transmissions, software and information technology services		
33	Finance		
34	Real estate		
35	Leasing and business services		
36	Scientific research and technical services		
37	Management of water conservancy, environment, and public facilities		
38	Services to households, repair, and other services		
39	Education		
40	Health care and social work activities		
41	Culture, sports, and entertainment		
42	Public management, social security, and social organization		
