# Supplementary material for: Mapping global carbon footprint in China
Source: Nat Commun. 2020 May 7;11:2237. doi: 10.1038/s41467-020-15883-9 (PMC7206023; doi:10.1038/s41467-020-15883-9)
Supplement: Supplementary file 3 — Description of Additional Supplementary Files [file 41467_2020_15883_MOESM3_ESM.pdf]

Description of Additional Supplementary File

File Name: Supplementary Data 1

Description: An example that shows the spatial footprint approach applied in this study. It has two parts: Part 1 on Data Construction and Part 2 on Emissions Hotspots.
